# Long-Term Follow-Up of Contrast-Induced Acute Kidney Injury: A Study from a Developing Country

**DOI:** 10.1155/2020/8864056

**Published:** 2020-12-21

**Authors:** Ashraf O. Oweis, Sameeha A. Alshelleh, Nesreen Saadeh, Mohamad I. Jarrah, Rasheed Ibdah, Karem H. Alzoubi

**Affiliations:** ^1^Division of Nephrology, Department of Medicine, Jordan University of Science and Technology, Irbid, Jordan; ^2^Division of Nephrology, Department of Medicine, The University of Jordan, Amman, Jordan; ^3^Department of Medicine, Jordan University of Science and Technology, Irbid, Jordan; ^4^Division of Cardiology, Department of Medicine, Jordan University of Science and Technology, Irbid, Jordan; ^5^Department of Clinical Pharmacy, Jordan University of Science and Technology, Irbid, Jordan

## Abstract

**Introduction:**

Contrast-induced acute kidney injury (CI-AKI) is a worldwide known complication related to the use of contrast media with either imaging or angiography; it carries its own complications and effect on both morbidity and mortality; early identification of patients at risk and addressing modifiable risk factors may help reducing risk for this disease and its complications.

**Methods:**

This was a prospective observational study, where all patients admitted for cardiac catheterization between June 2015 and January 2016 were evaluated for CI-AKI. There were two study groups: contrast-induced acute kidney injury (CI-AKI) group, and noncontrast-induced acute kidney injury (non-CI-AKI) group.

**Results:**

Patients (*n* = 202) were included and followed up for 4 years. Death and development of chronic kidney disease (CKD) need for another revascularization were the end points. The incidence of CI-AKI was 14.8%.In univariate analysis, age (*P* = 0.016) and serum albumin at admission (*P* = 0.001) were statistically significant predictors of overall death. Age (*P* = 0.002), HTN (*P* = 0.002), DM (*P* = 0.02), and the use of diuretics (*P* = 0.001) had a statistically significant impact on eGFR. The rate of recatheterization was not statistically significant between the two groups (61 (35.5%) for the non-CI-AKI vs. 12 (40%) for the other group; *P* = 0.63). Some inflammatory markers (NGAL *P* = 0.06, IL-19 *P* = 0.08) and serum albumin at admission *P* = 0.07 had a trend toward a statistically significant impact on recatheterization. Death (*P* = 0.66) and need for recatheterization (*P* = 0.63) were not statistically different between the 2 groups, while the rate of eGFR decline in for the CI-AKI was significant (*P* = 0.004).

**Conclusion:**

CI-AKI is a common complication post percutaneous catheterization (PCI), which may increase the risk for CKD, but not death or the need for recatheterization. Preventive measures must be taken early to decrease the morbidity.

## 1. Introduction

Contrast-induced acute kidney injury (CI-AKI) is a potentially preventable and reversible cause of acute kidney injury (AKI). Multiple factors are associated with the development of CI-AKI, some are modifiable such as drugs, type, and amount of contrast media used, and others are not such as preexisting chronic kidney disease (CKD) or heart failure [1].

Though it is usually a reversible condition, there is increasing evidence of adverse long-term outcome including increased morbidity and mortality [2]. Identifying risks and adapting some protocols may help decreasing the incidence and impact of this disease [3, 4]. It is apparent that CI-AKI contributes to long-term mortality, revascularization need, and increased risk for CKD [4, 5].

The incidence of CI-AKI has been variable among studies (2-15%), and the definition to be used for CI-AKI is still debatable, whether to use AKIN, RIFLE with MDRD, or RIFLE with CKD-EPI or CK or KIDOGI is also variable between studies [6]. According to a large cohort of patients who underwent PCI post-STEMI, the RIFLE with CKD-EPI was the most to determine and predict complications like inhospital mortality, one-year mortality, and need for dialysis at 1 year [7]. In general, the incidence of CI-AKI post-PCI can differ even on the same cohort according to the definition used for CI-AKI [8]. The aim of this study is to evaluate the long-term outcome of CI-AKI on mortality, the rate of recatheterization, and the development of CKD in a health setting of a developing country.

## 2. Method

This study was a prospective observational study. All patients admitted for cardiac catheterization of King Abdulla University Hospital, Irbid, Jordan, over a period of 6 months were included. This tertiary hospital is the largest hospital at north province of Jordan with over 700 beds capacity. This study had 326 patients as participants and represents a secondary analysis of our previous study [9]. Every participant had to sign the consent form. For the purpose of measuring creatinine levels, blood was withdrawn 48 hours after the procedure.

The CI-AKI was defined as >25% or 44 mmol/l increase in serum creatinine from the baseline level by 48-72 hours—without any other obvious cause—after administration of contrast media [10–12]. The Modification of Diet in Renal Disease (MDRD) equation was used to estimate glomerular filtration rate (eGFR). Low-osmolality contrast media (CM) was the only used media among patients in the current study (Lopamidol, Bayer, Germany, 616 mOsm/kg H_2_O). Only 202 patients of the original 326 patients included had a second sample withdrawn, thus, were qualified to remain in the study. There were two study groups: the contrast-induced acute kidney injury (CI-AKI) group and noncontrast-induced acute kidney injury (non-CI-AKI) group. Patients were followed for 4 years; data for long-term follow-up was obtained either from the hospital records or phone calls. The primary endpoints were mortality, development of CKD, and the need for recatheterization by the end of follow-up. This study protocol was approved by the institutional review board of Jordan University of Science and Technology. Written informed consent forms were obtained from all study subjects.

### 2.1. Cytokines Measurements

Serum samples were stored at −80°C in aliquots until the time of analysis. The levels of IL-1*α*, IL-6, IL-19, IL-20, IL-21, IL-22, and IL-33 were directly measured—without dilution—from serum, whereas the levels of NGAL were done after 1 : 20 dilution. Measurement was carried out according to commercial kits as per the manufacturers' instructions (IL-1*α* (R&D Systems, Inc., Minneapolis, MN, USA), IL-6 (R&D Systems), IL-19 (R&D Systems), IL-20 (R&D Systems), IL-21 (eBioscience, San Diego, CA, USA), IL-22 (R&D Systems), IL-33 (R&D Systems), and NGAL (RayBio, Norcross, GA, USA). The samples in reaction mixtures were read at 450 nm wavelength.

### 2.2. Statistical Analysis

Analysis was performed using STATA/MP, version 14.0 (StataCorp LLC, College Station, TX, US). Data were described using percentages for categorical variables and means and SD for continuous variables. There were two study groups in the current study: patients with CI-AKI and those without CI-AKI. To compare the means in continuous variables, *t*-test for independent samples was performed. Chi-squared test was used to test the association between the demographic, clinical, and other relevant characteristics of the participants and the incidence rates of CI-AKI (with and without CI-AKI). To determine the influence of factors associated with CI-AKI, multivariate binary logistic regression was done.

## 3. Results

**Figure 1:**
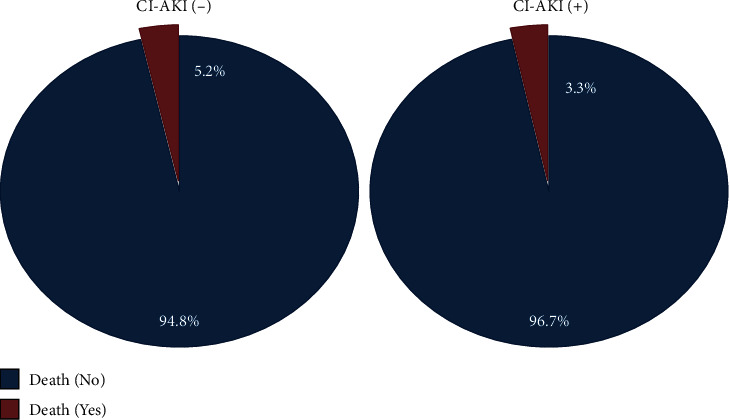
Mortality based on CI-AKI status.

**Figure 2:**
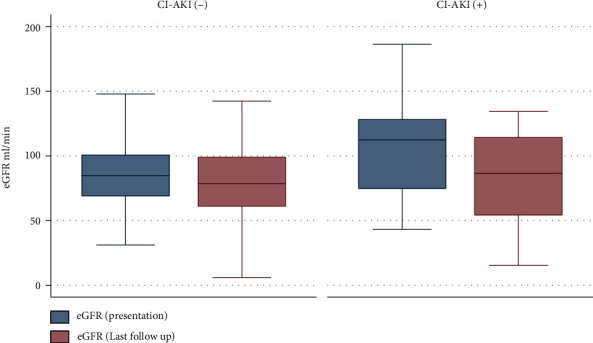
eGFR by CI-AKI status.

The incidence of CI-AKI was 14.8% (30 patients). Patients' age was 55.8 ± 9.7 years, 28.2% of them were females, and 54.2% of them were either current or past smokers. Additionally, 43.6% of the patients' diabetes mellitus, 68.8% had hypertension, 26.8% had congestive heart failure, 10.4% had cerebrovascular accident, and 47.0% had coronary artery disease. At the end of follow-up, a total of 10 patients died, nine in the non-CI-AKI group vs. one in the CI-AKI group (*P* = 0.66); the mean time to death was 22.1 months (SD ± 14.1; Figure 1). In univariate analysis, age (*P* = 0.016) and serum albumin at admission (*P* = 0.001) were statistically significant predictor of overall death, while patients' gender, underlying comorbidities, drugs, and different inflammatory markers (IL-1, IL-6, IL-19, IL-20, Il-21, IL-22, IL-33, and CRP) did not have an impact on mortality. Although the differences between the mean eGFRs, by the end of the follow-up period, were not statistically significant (85.4 ml/min for the CI-AKI vs. 79.2 ml/min for the other group (*P* = 0.31)), decline in eGFR for the CI-AKI was significant when pre- versus post-eGFRs were compared. A drop from 105.4 ml/min to 85.4 ml/min vs. 85.2 ml/min to 79.2 ml/min, *P* = 0.004; Figure 2. Age (*P* = 0.002) but not gender (*P* = 0.34) was associated with worsening eGFR. HTN (*P* = 0.002), DM (*P* = 0.02), and the use of diuretics (*P* = 0.001) had a statistically significant impact on eGFR.

Mean time to recatheterization was 17.5 months (SD ± 12.5); 15.8 months for the CI-AKI group (SD ± 13.9) vs. 17.8 months for the other group (SD ± 12.3), *P* = 0.61. The rate of recatheterization was not statistically significant between the two groups (61 (35.5%) for the non-CI-AKI vs. 12 (40%) for the other group; *P* = 0.63). As expected, history of CAD (*P* = 0.001) and the use of statins (*P* = 0.004) had an effect on the rate of recatheterization, while age, gender, comorbidities, and drugs did not. Patients with more than one stent inserted were more likely to undergo recatheterization (*P* = 0.003). Some inflammatory markers (NGAL—*P* = 0.06, IL19—*P* = 0.08) and serum albumin at admission (*P* = 0.07) had a trend toward statistically significant impact on recatheterization, see Table 1.

## 4. Discussion

In the current study cohort, the incidence for CI-AKI was 14.8%, which was close to the results of the cohort by Uzunhasan et al., where CI-AKI happened in16.4% of patients who underwent catheterization for ACS [5]. Having one or multiple vessels are catheterized during the PCI postcardiac event no effect on incidence of CI-AKI [13, 14]. Predictors for CI-AKI were as follows: age, HTN, DM, and the use of diuretics. As expected, with aging, multiple factors can affect negatively influence kidneys and decrease eGFR such as increase in oxidative stress, decrease in local nitric oxide, decreased renal plasma flow, atherosclerotic changes, and damage to filtration barriers. A recent meta-analysis review done by Morcos et al. showed age as risk for CI-AKI; factors like hypertension, DM, contrast volume, and anemia will increase risk too [1]. Thrombolysis in myocardial infarction (TIMI) Risk Index (TRI) was found to be as independent risk factor for CI-AKI in patients who underwent PCI for ACS [15].

Serum albumin precatheterization can increase the risk for CI-AKI due to its relationship with endothelial dysfunction and background inflammatory response. This was shown in a cohort by Murat et al. in patients who underwent PCI for ACS, where serum albumin level was also a risk for CI-AKI [16]. Prealbumin levels can be associated with increased risk for CI-AKI and long-term mortality in elderly undergoing PCI [17].

Other biomarkers like NGAL are noticed to increase with CI-AKI especially with advanced stages of CKD urinary NGAL can predict the risk and severity of CI-AKI [18, 19]. Still, some studies did not show a significant predictive value of serum NGAL that allows prediction of CI-AKI in patients undergoing PCI post-ST elevation myocardial infarction (STEMI) [20]. Other markers in urine and blood were also studied in trial to predict and diagnose CI-AKI early enough to reduce complications [21].

The CI-AKI did not affect mortality in the current cohort. The incidence for mortality was 3.3% in CI-AKI group vs. 5.2% in non-CI-AKI group with mean time to death 22.1 months (SD ± 14.1). At the same time, the difference between these two groups in the degree of eGFR drop or change was not statistically significant. These findings regarding mortality and change in renal function correlate well with findings from Ribitsch et al. cohort where no difference in creatinine was noticed or mortality by 2 years follow-up [2].

Baseline kidney function, being a CKD patient, may not affect the risk to develop CI-AKI but has its effect on both inhospital and long-term mortality post PCI. Even risk for future cardiovascular adverse events like STMI, NSTEMI, and stroke were higher in patients with CI-AKI [22, 23]. Worse long-term outcomes were noticed in patients who underwent PCI for left main coronary artery stenosis with cardiogenic shock too [24]. In general, the length of hospital stay specifically decreased with adapting PCI if compared with patients with ACS and no PCI, risk for longer hospital stay were older age, female gender, and pacific ethnicity in addition to no PCI or need for CABG [25]. Short-term post-PCI complications such as leading to hospitalization and increased ninety days mortality are expected to be higher in patients with incomplete revascularization and with TIMI flow <3 at end of PCI especially for the left main coronary artery [26].

Regarding the need for another catheterization for revascularization, nine RCTs were studied by Xu et al. showed that complete revascularization if compared with infarct-related coronary artery revascularization only was associated with lower cardiac complications such as lower rate of revascularization, lower cardiac events, lower cardiac death, and all-cause mortality. It was also worth to mention that rate of CI-AKI was not different between both groups [27]. Others also showed superiority of complete revascularization over infarct-related coronary artery revascularization [28].

In CKD patients, the risk for incomplete revascularization is higher and as expected is associated with higher complications and worse outcomes [29]. In some studies, the tendency of recatheterization for revascularization is lower in CKD patients if compared with others [30, 31].

We found that comorbidities did not affect the rate for recatheterization for revascularization in our cohort, which is relatively small. Still, being a patient with diabetes will have a constant risk for ACS and its complications and fatality if compared with others, which their risks decreased over time with introduction of revascularization procedures [32]. Loading dose of statins prerevascularization was found to be associated with lower risk of CI-AKI and complications [33, 34]. In our cohort, the use of statins was also associated with lower rate for events and revascularization.

We evaluated additional factors like serum interleukins; we found that IL-1 has an effect on mortality as outcome but not revascularization. IL-1 may initiate an inflammatory response and increase atherosclerosis especially in CKD patients [35]. The IL-19 may play some role in the rate of revascularization. This may be due to the effect of IL-19 effect on enhancing angiogenesis by macrophage polarization and decrease risk for atherosclerosis [36].

In conclusion, CI-AKI is a significant contributor to both short-term and long-term complications post-PCI. Efforts must be taken to identify patients at risk for CI-AKI, which may decrease cardiac morbidity and mortality, risk for CKD, and rate of revascularization.

## Figures and Tables

**Table 1:** Effect of different markers on outcomes.

Marker	Death (*P* value)	CKD (*P* value)	Recatheterization (*P* value)
IL1	**0.03**	0.38	0.39
IL6	0.41	0.18	0.30
IL19	0.47	0.25	**0.08**
IL20	0.93	0.85	0.85
IL21	0.33	0.68	0.22
IL22	0.79	0.50	0.40
IL33	0.91	0.34	0.80
NGAL	0.19	0.65	**0.06**
CRP	0.93	0.20	0.28
S. albumin	**0.001**	0.15	**0.07**
Hb	0.15	**0.06**	0.37
Vitamin D	0.87	0.67	0.94

## Data Availability

Data will be available upon request via e-mailing the corresponding author.
